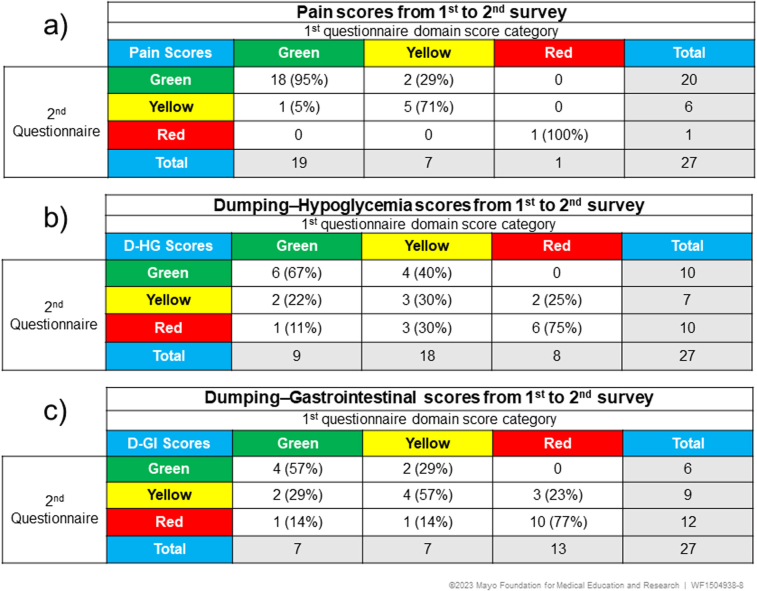# Patients Struggle With Severe Symptoms Even After Surviving Esophagectomy for Esophageal Cancer

**DOI:** 10.1016/j.atssr.2023.09.010

**Published:** 2023-11-03

**Authors:** Mohamad K. Abou Chaar, Anny Godin, Sahar A. Saddoughi, William M. Harmsen, Minji K. Lee, Kathleen J. Yost, Shanda H. Blackmon

**Affiliations:** 1 Division of Thoracic Surgery, Department of Surgery, Mayo Clinic, Rochester, Minnesota; 2 Department of Biostatistics, Mayo Clinic, Rochester, Minnesota; 3 Kern Center for the Science of Health Care Delivery, Mayo Clinic, Rochester, Minnesota; 4 Department of Health Sciences Research, Mayo Clinic, Rochester, Minnesota; 5 Center for Digital Health, Mayo Clinic, Rochester, Minnesota

## Abstract

**Background:**

This prospective study was conducted to determine postesophagectomy symptom severity of esophageal cancer survivors with use of the Upper Digestive Disease (UDD) questionnaire.

**Methods:**

A prospective trial included adult esophagectomy patients diagnosed with primary esophageal carcinoma at a single institution from 2000 to 2011. Those who remained alive in 2015 to 2021 were enrolled. Comparison was made between sequential questionnaires.

**Results:**

From a prospective registry of 895 patients with esophagectomy for esophageal cancer, 297 (33%) long-term survivors were identified. Of those, 93 (31%) had recent contact data and 66 (71%) consented and completed the UDD questionnaire. Most participants, 77% (51), were men with a mean age of 57 (±7) years. The mean time from esophagectomy was 12 (8-20) years. The 66 enrolled patients completed 127 UDD questionnaires. A total of 27 (41%) completed at least 2 questionnaires. Poor performance was recorded in the 5 domains as follows: reflux, 19 patients (29%); pain, 3 patients (5%); dysphagia, 0 patients; gastrointestinal dumping, 31 patients (47%); and generalized dumping, 17 patients (26%). Between the first and second questionnaires, dysphagia had the most noticeable improvement in domain score (23/27 [85%]), and reflux had the most regression in domain score (7/27 [26%]).

**Conclusions:**

Patient-reported outcome data are an integral part of esophageal cancer survivorship care. Having a standardized tool that would enhance research and standardize care pathway symptom management is needed.

## Visual Abstract

**Figure:**
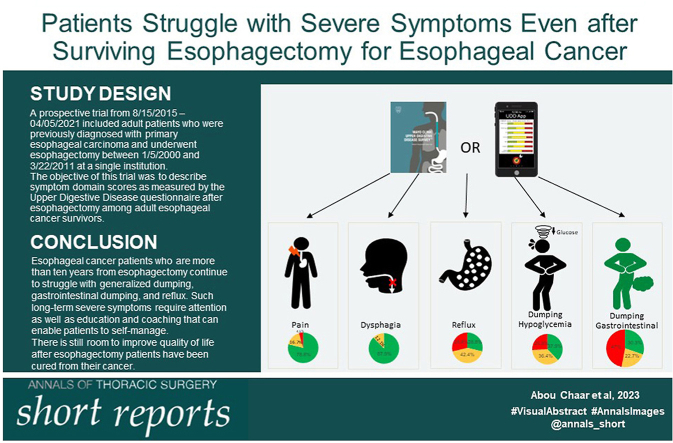

**In Short**

▪ Esophagectomy patients who are cured of esophageal cancer continue to struggle with generalized dumping, gastrointestinal dumping, and reflux.
▪ Even after a decade, such long-term severe symptoms require attention.

Survival from esophageal cancer has improved in the last decade as a result of advances in staging, systemic treatment, and minimally invasive surgical resection.^1^ Long-term survivors continue to struggle with impaired quality of life after recovering from the immediate perioperative phase.^2^ Patient-reported outcomes (PROs), including health-related quality of life, are also related to overall survival.^3^

The Mayo Clinic Upper Digestive Disease (UDD) questionnaire is a patient-centered tool comprising multi-item symptom assessment domains and health assessment domains.^4^ The primary objective of this study was to measure PROs from esophagectomy for primary esophageal cancer to determine what percentage of long-term survivors continue to struggle with symptoms and help us design interventions in the future.

## Material and Methods

A prospective nonrandomized study recorded PROs after curative esophagectomy for primary esophageal cancer in a high-volume single institution between August 15, 2015, and April 5, 2021, after institutional review board approval (14-009873). Patients older than 18 years undergoing esophagectomy from January 1, 2000, to March 22, 2011, were identified. Only those who survived >8 years after esophagectomy with contact information were approached for consent. Patients were considered lost to follow-up if they were approached on 3 consecutive events with no response. Long-term survivor was the term used to describe any patient who survived beyond 5 years of curative after esophagectomy. A digital or paper version of the UDD questionnaire was administered according to the patient’s preference. Only the first results were included for analysis of risk factor association with domains. A separate analysis was done to detect change in domain score on the basis of patient self-management. Providers were not notified about scores. Only the UDD digital version provided patients with color-coded score dashboards.

From a prospectively maintained database, demographic characteristics and disease information were collected. All esophageal cancer staging was based on the American Joint Committee on Cancer seventh edition guidelines. Esophagectomies were performed in Mayo Clinic, Rochester, Minnesota. Surgical approach was based on tumor location. Only type III-IV anastomotic leaks were included.^5^ Vital and recurrence status was collected.

Change in questionnaire score was calculated as second questionnaire minus first questionnaire; therefore, a positive change indicates a higher second questionnaire score number. A test of the change being non-0 was made by a Wilcoxon signed rank test. Correlation between changes in domain score was estimated with a Spearman rank correlation coefficient. Associations between patient and disease variables and score severity status were done by logistic regression models. Recurrence and disease-free survivals were analyzed by the Kaplan-Meier survival method. The α level was set at .05 for statistical significance. SAS software version 9.4 (SAS Institute) was used for analyses.

## Results

**Supplemental Figure 1:**
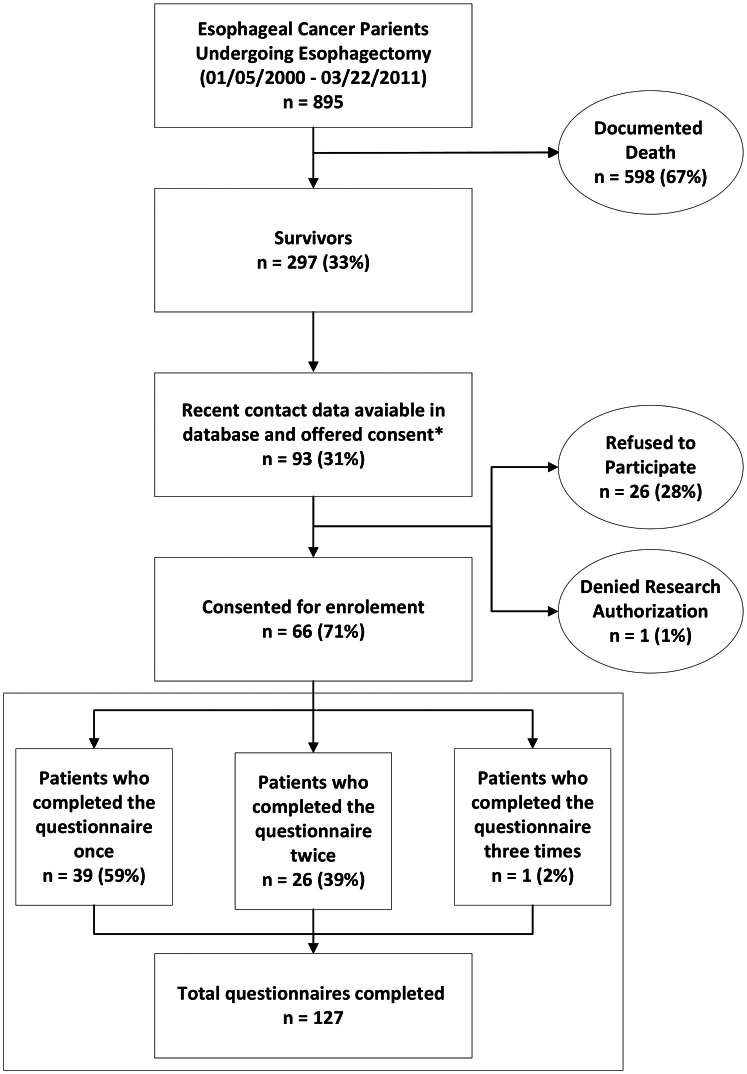


During the study period, 297 of 895 (33%) esophagectomy survivors were identified. Viable contact information was available for 93 of 297 patients. When contacted, a total of 66 of 93 (71%) provided consent to participate (Supplemental Figure 1). Most patients were men (51/66 [77%]), all of whom were White, with a mean age of 57.2 (±7.4) years (Table 1).

**Table 1:** Characteristics of Patients From Study Cohort

Variables	(N = 66)
Age at surgery, y	
Range	41-76
Median (Q1-Q3)	57 (52.7-62.3)
Sex	
Male	51 (77)
Female	15 (23)
Comorbidities	
Diabetes mellitus	6 (9)
Coronary artery disease	6 (9)
Renal disease	1 (2)
Smoking history	
Yes	11 (17)
No	25 (38)
Ex-smoker	29 (44)
Alcohol use	
Yes	44 (67)
No	16 (24)
Previous user	4 (6)
Cancer type	
Adenocarcinoma	57 (86)
Squamous cell carcinoma	9 (14)
Neoadjuvant treatment	
Yes	37 (56)
No	29 (44)
Surgical approach^a^	
Open	65 (98.5)
Minimally invasive	1 (1.5)
Adjuvant treatment	
Yes	7 (10.6)
No	59 (89.4)
Pathologic stage	
Missing	1
0	13 (20)
IA/IB	19 (29.2)
IIA/IIB	27 (41.5)
IIIA/IIIB/IIIC	5 (7.7)
IV	1 (1.5)
Postoperative complications	
Anastomotic leak (grade 3-4)	0
Chyle leak	4 (6)
Surgical reintervention	5 (8)
Respiratory failure	0
Infection	13 (20)
Atrial fibrillation	5 (8)
Transfusion	22 (33)
Renal failure	2 (3)
Other^b^	8 (12)

Categorical variables are presented as number (percentage). Continuous variables are presented as median (interquartile range).

a Almost all patients underwent pyloroplasty/pyloromyotomy with a gastric tube conduit except 1 patient, who had a total gastrectomy with Roux-en-Y.

b This included vocal cord paralysis, escalation of care, and readmission.

Of the 66 patients with esophageal cancer who underwent curative esophagectomy and then completed at least 1 UDD questionnaire, 127 questionnaires were submitted, reviewed, and scored. A total of 26 patients (39%) completed the questionnaire twice without prompting, and only 1 (2%) completed 3 questionnaires. Poor, moderate, and good domain scores are shown in the Figure and Table 2. There was no association between the severity of the score in any domain and any clinical factor. Two-thirds of patients who reported severe pain pointed out that their pain was localized at or around the incision site (indicating post-thoracotomy pain syndrome).^6^

**Figure:**
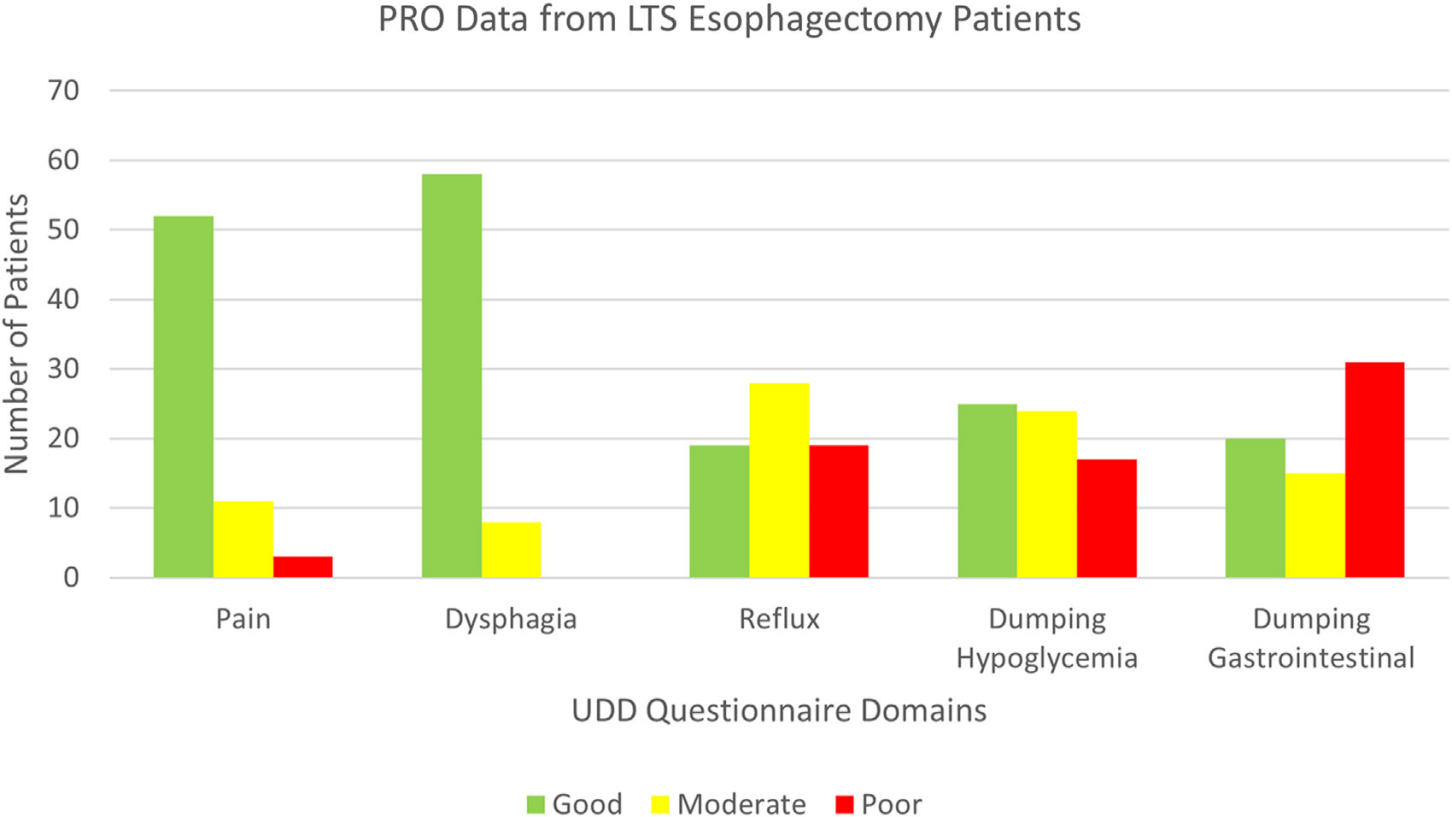
Column chart detailing the numbers of long-term survivor (LTS) esophagectomy patients with patient-reported outcome (PRO) scores according to the Upper Digestive Disease (UDD) questionnaire domain (pain, dysphagia, reflux, dumping hypoglycemia, dumping gastrointestinal) and category (green, good; yellow, moderate; red, severe).

**Table 2:** Distribution of Domain Scores Divided by Category (Good, Moderate, Severe) From the First Survey Taken

Domain	Score (N = 66)
Pain	
Good	52 (78.8)
Moderate	11 (16.7)
Poor	3 (4.5)
Dysphagia	
Good	58 (87.9)
Moderate	8 (12.1)
Poor	0
Reflux	
Good	19 (28.8)
Moderate	28 (42.4)
Poor	19 (28.8)
Dump hypoglycemia	
Good	25 (37.9)
Moderate	24 (36.4)
Poor	17 (25.8)
Dump gastrointestinal	
Good	20 (30.3)
Moderate	15 (22.7)
Poor	31 (47.0)

Values are reported as number (percentage).

To illustrate the change in domain scores over time of the 27 patients who completed multiple entries, we compared the first and second questionnaires (Supplemental Figure 2). For pain, a total of 19 patients were initially found to have good overall score, 1 (0.5%) of whom regressed to poor in the second questionnaire. Change in dysphagia was minimal.

Half of those who scored within the good zone for reflux in the first questionnaire (4/8 [50%]) were found to have a score in the moderate zone in the second one, and 3 of 14 (21%) patients regressed from moderate to severe. Of patients who initially scored in the moderate zone, 5 of 14 (36%) improved to good, and 1 of 5 (20%) improved from severe to moderate (Supplemental Figure 2a).

For hypoglycemic dumping, 4 of 10 (40%) patients who were in the moderate zone transitioned to good, and 2 of 8 (25%) patients who were in the severe zone transitioned to moderate. However, 3 of 10 (30%) of those who started in the moderate zone worsened to severe; 2 of 9 (22%) who started in the good zone worsened to moderate and 1 of 9 (11%) worsened to severe (Supplemental Figure 2b).

Changes in the gastrointestinal dumping domain scores were as follows: 2 of 7 (29%) patients who started in the moderate zone improved to good; 3 of 13 (23%) who started in the severe zone improved to moderate; 1 of 7 (14%) who started in the good zone worsened to severe; 1 of 7 (14%) who started in the moderate zone worsened to severe; and 2 of 7 (29%) who started in the good zone worsened to moderate (Supplemental Figure 2c).

The only variable with statistically significant change over time was the Patient-Reported Outcomes Measurement Information System (PROMIS) physical function score (*P* = .018), which demonstrated poorer physical health overall on subsequent surveys (Table 3). A significant association between change in pain and dysphagia scores was found with a Spearman correlation coefficient *r* of 0.5 and *P* value of .012, and another significant association was identified between change in gastrointestinal dumping level and pain level (*r* = 0.4; *P* = .038). No significant association was found between any of the other domain scores or levels over time.

**Table 3:** Wilcoxon Signed Rank Test Showing Change in Scores From First to Second Survey in all Five Domains in Addition to the Two PROMIS Scores

Change in:	Total (N = 27)	*P* Value	Overall Status
PROMIS physical^a^		.0175^b^	Regression
Mean (SD)	−2.1 (4.3)		
Median	−2.8		
Q1-Q3	−5.4 to 0.0		
Range	−11.1 to 10.0		
PROMIS mental^a^		.2360^b^	Regression
Mean (SD)	−1.4 (5.1)		
Median	0.0		
Q1-Q3	−3.0 to 0.0		
Range	−16.8 to 9.7		
Pain score		.4795^b^	Improvement
Mean (SD)	1.1 (13.2)		
Median	0.0		
Q1-Q3	−0.0 to 11.7		
Range	−41.1 to 23.5		
Pain level^c^		1.0^b^	
Improvement	2 (7.4%)		
No change	24 (88.9%)		
Regression by 1 level	1 (3.7%)		
Dysphagia score		.1166^b^	Improvement
Mean (SD)	5.1 (13.7)		
Median	0.0		
Q1-Q3	0.0, 8.6		
Range	−20.0 to 39.5		
Dysphagia level^c^		.1250^b^	
Improvement	23 (85.2%)		
No change	4 (14.8%)		
Reflux score		.1240^b^	Improvement
Mean (SD)	5.6 (16.6)		
Median	5.9		
Q1-Q3	−8.8 to 17.1		
Range	−18.9 to 40.2		
Reflux level^c^		1.0 b	
Improvement	6 (22.2%)		
No change	14 (51.9%)		
Regression by 1 level	7 (25.9%)		
Dumping hypoglycemia score		.7298^b^	Improvement
Mean (SD)	0.1 (18.0)		
Median	0.0		
Q1-Q3	−6.7 to 5.9		
Range	−33.3 to 50.7		
Dumping hypoglycemia level^c^		1.0^b^	
Improvement	6 (22.2%)		
No change	15 (55.6%)		
Regression by 1 level	5 (18.5%)		
Regression by 2 levels	1 (3.7%)		
Dumping gastrointestinal score		.8056^b^	Regression
Mean (SD)	−0.6 (20.9)		
Median	0.0		
Q1-Q3	−18.1 to 9.1		
Range	−36.4 to 45.5		
Dumping gastrointestinal level^c^		1.0^b^	
Improvement	5 (18.5%)		
No change	18 (66.7%)		
Regression by 1 level	3 (11.1%)		
Regression by 2 levels	1 (3.7%)		

PROMIS, Patient-Reported Outcomes Measurement Information System.

a Change in domain score overall.

b Wilcoxon signed rank test.

c Changing domain color-coded category over time.

## Comment

We identified that more than a third of esophagectomy survivors continue to struggle with severe gastrointestinal dumping, hypoglycemic/systemic dumping, and reflux long after they are cured of esophageal cancer. In the domains that are easiest to diagnose and to manage, such as pain and dysphagia, improved scores are more frequently reported. Once patients with esophageal cancer are beyond 8 to 20 years from multimodality treatment, they tend to have little or no improvement in symptoms when left to self-manage.

Collecting PROs alone is not adequate to address symptoms. However, the addition of dashboard feedback, targeted patient education for moderate symptoms, and provider-based intervention for poor symptoms can improve scores over time.^7^ Studies indicate that improving domain scores can also improve associated quality of life scores.^7^^,^^8^

Limitations to this study include its nonrandomized design, small sample size, conduct at a single institution, and wide window from surgery at the time of initial enrollment. Although there were very few missing data, a significant proportion of patients were lost to follow-up over time. Clustering of symptoms was not seen in this cohort, but larger numbers would be required to detect true clustering.

Esophageal cancer survivors after esophagectomy need curated tools to improve their health-related quality of life outcomes even when they are >5 years after surgery. This study was the first in a stepwise approach to provide meaningful and impactful tools to identify these problems.^4^

## Figures and Tables

**Supplemental Figure 2:**