# Supplementary material for: Patients Struggle With Severe Symptoms Even After Surviving Esophagectomy for Esophageal Cancer
Source: Ann Thorac Surg Short Rep. 2023 Nov 3;2(1):98–102. doi: 10.1016/j.atssr.2023.09.010 (PMC11708654; doi:10.1016/j.atssr.2023.09.010)
Supplement: Legend for Supplemental Figures 1 and 2 [file mmc1.docx]

**Supplementary Figures:**

**Supplementary Figure 1.** Column chart showing the survival percentage for esophageal cancer from 1975 to 2019 according to the SEER Database.

**Supplementary Figure 2.** Numbers of patients according to domain who took 2 surveys and the change in score from the first to the second time the questionnaire was taken to demonstrate distribution of score change in a) pain, b) dumping-hypoglycemia, c) dumping-gastrointestinal over time.
